# Urea and creatinine detection on nano-laminated gold thin film using Kretschmann-based surface plasmon resonance biosensor

**DOI:** 10.1371/journal.pone.0201228

**Published:** 2018-07-27

**Authors:** P. Susthitha Menon, Fairus Atida Said, Gan Siew Mei, Dilla Duryha Berhanuddin, Akrajas Ali Umar, Sahbudin Shaari, Burhanuddin Yeop Majlis

**Affiliations:** Institute of Microengineering and Nanoelectronics, Universiti Kebangsaan Malaysia, UKM Bangi, Selangor, Malaysia; Institute of Materials Science, GERMANY

## Abstract

This work investigates the surface plasmon resonance (SPR) response of 50-nm thick nano-laminated gold film using Kretschmann-based biosensing for detection of urea and creatinine in solution of various concentrations (non-enzymatic samples). Comparison was made with the presence of urease and creatininase enzymes in the urea and creatinine solutions (enzymatic samples), respectively. Angular interrogation technique was applied using optical wavelengths of 670 nm and 785 nm. The biosensor detects the presence of urea and creatinine at concentrations ranging from 50–800 mM for urea samples and 10–200 mM for creatinine samples. The purpose of studying the enzymatic sample was mainly to enhance the sensitivity of the sensor towards urea and creatinine in the samples. Upon exposure to 670 nm optical wavelength, the sensitivity of 1.4°/M was detected in non-enzymatic urea samples and 4°/M in non-enzymatic creatinine samples. On the other hand, sensor sensitivity as high as 16.2°/M in urea-urease samples and 10°/M in creatinine-creatininase samples was detected. The enhanced sensitivity possibly attributed to the increase in refractive index of analyte sensing layer due to urea-urease and creatinine-creatininase coupling activity. This work has successfully proved the design and demonstrated a proof-of-concept experiment using a low-cost and easy fabrication of Kretschmann based nano-laminated gold film SPR biosensor for detection of urea and creatinine using urease and creatininase enzymes.

## Introduction

End stage renal disease (ESRD) or commonly known as kidney failure is one of the major public health problems affecting approximately 10% of the world population. Imminently, the number of the ESRD patients that require haemodialysis continue to rise mainly due to the increase in cases of hypertension and diabetes and also in the growing elderly population. Kidney failure is often manifested by the unusual albumin secretion and decreased kidney function. During pathophysiological conditions, urea concentration ranges from 30–150 mM while the normal level range is between 2.5–6.7 mM [1]. The pathophysiological level of creatinine may rise up to >1000 μM whereas the normal level of creatinine in serum/plasma is 35–140 μM [2]. The high level of creatinine indicates diabetic nephropathy, preeclampsia, glomerulonephritis, urinary tract obstruction, and renal failure, whereas low levels indicate muscular dystrophy and myasthenia [2,3]. Haemodialysis treatment helps patients with kidney failure to filter out urea and creatinine from the blood [4]. Consequently, it is important to quantify and monitor the level of urea and creatinine results from haemodialysis procedure to evaluate the effectiveness of the haemodialysis treatment.

Haemodialysis monitoring system may include an optical sensor to detect urea and creatinine as the kidney wastes. The surface-enhance Raman scattering (SERS) which is one of the optical sensing method for urea and creatinine detection have been previously investigated. Leordean and coworkers [5] reported a direct and reliable identification of urea metabolic by-product in tears, fingerprints, and urine by SERS using gold colloidal particulate films as SERS active substrate. Bakar et al. [6] explore detection of creatinine concentration on a triangular silver nanoplate films surface using self-build SERS sensor More recently, Abdullah and coworkers [7] investigated detection of creatinine using silver-platinum nanoferns substrates for a SERS sensor fabrication. The SERS has proven to be a label-free, highly sensitive, and selective sensing method through enhanced localized electric field (‘hot spot’) on the surface of colloidal plasmonic structure when excited with monochromatic light [8–10]. However, this method could suffer from spatial inhomogeneities in the Raman enhancement due to random distribution of the colloidal nanostructures [11]. Despite the high SERS enhancement factor using colloidal nanostructure substrate, the intensity of the SERS signal fluctuates due to Brownian motion and diffusion of clusters through the scattering volume during solution phase measurement. In case of solid phase analysis, irregular ‘hot spot’ generated by aggregated colloids on the dried surface could occur [12]. For these reasons, colloidal SERS substrates is challenging for the use in quantitative measurement.

Alternatively, the Kretschmann-based surface plasmon resonance (SPR) sensing approach using planar thin metal film offers distinct advantages over other label-free sensing techniques. Firstly, planar layer of high quality and uniformity are readily manufactured. A wide range of commercial SPR sensor slides is available in the market which give remarkably low limit of detection. Secondly, the field confinement resulting from the excitation of propagating surface plasmon polariton is highly uniform over large areas, albeit with weaker field confinement than the localized surface plasmon polariton counterpart using metal nanostructures [13]. Adding to that, the well-established theoretical treatment of planar surface by rigorous solution to Maxwell’s equation enables accurate comparison between theoretical and experimental studies [11]. For instance, Jamil et al. [14] numerically investigated detection of urea on Kretschmann based SPR biosensor using sensor slide made of molybdenum disulphide, graphene, gold, and aluminium oxide layers. It showed that Kretschmann-based SPR sensing technique have potential for application in urea and creatinine detection.

The SPR phenomenon, in principle, is due to the confined electromagnetic wave of surface plasmon polariton in the vicinity of dielectric-metal interface. In fact, the Kretschmann configuration of the attenuated total reflection method is frequently used for SPR excitation [15]. It consists of a high refractive index dielectric prism, a surface plasmon active metal layer, and analyte sensing surface. In this configuration, p-polarised light (TM mode) illuminates on the metal surface through the prism to excite the surface plasmon wave at metal-dielectric interface [16]. When the incident angle of the light source fulfils the SPR condition, the propagating constant of incident light along the interface will match that of surface plasmon wave causing the collective oscillation of electrons on the surface of the metal [17]. In this state the energy from incident light transfer onto the surface plasmons leads to significant decrease in the reflectivity and forms a sharp dip in the SPR response curve. Hence, the SPR occurrence is represented by a sharp dip in the SPR response curve which indicates the minimum reflectivity based on the total internal reflection at the metal-dielectric interface. The SPR excitation are strongly sensitive to the changes of refractive index on the sensing surface, and therefore the shift in the incident angle (in angular interrogation mode) or the optical wavelength (in wavelength interrogation mode) indicate presence of analyte in the sample [18]. The SPR technique has received continuous interest in various applications involving hazardous gases [19–21], heavy metal ions [22,23], drug molecules [24], multi-analytes [18,25], glucose [26] and immuno-sensing [27], and biomolecular binding studies [28] due to its advantages of real-time and label-free sensing technique.

The urea and creatinine sensing research has been explored in various techniques such as ion sensitive field effect transistors, optical fibre-based SPR, and electrochemical system. Bhatia et al. [29] reported a sensitivity of 0.2 nm/mM for the detection of urea using plasmonic optical fibers and wavelength interrogation method with 40 nm thick of Ag and 8 nm thick silica. Tran at el [30] reported a sensitivity of 685 μAmM^-1^cm^-2^ for the detection of urea using multi-wall carbon nanotubes with the presence of Ni-MOF. Finally, Raveendran et al [31] disclosed a sensitivity limit of 0.0746 μM for a electrochemical biosensor utilizing copper for the detection of creatinine. In spite of these, detection of urea and creatinine using Kretschmann-based SPR biosensor along with urease and creatininase enzyme has not yet been reported in the literature.

In this paper, we propose an SPR-based sensor using Kretschmann configuration with urea-urease and creatinine-creatininase enzymatic sample that offers a simple, economical and label-free method of detection with excellent sensitivity. Urea and creatinine solutions of various concentrations in non-enzymatic samples as well as enzymatic samples were analysed by measuring the changes in refractive index of the samples at optical wavelengths of 670 and 785 nm. In brief, the commercially available Lumerical’s finite difference time domain (FDTD) software was used to find the optimum incident wavelength and thickness of the nano-laminated gold film. Afterward, these parameters were used for experimental investigation. This work corresponds to the early stages of designing a Kretschmann-based SPR biosensors for medical applications in artificial kidney. Specifically, the SPR technique can be used as a sensing method for determining the adequacy of dialysis using determination of urea and creatinine concentration in the dialysate effluent from artificial kidney. The knowledge can be subsequently employed in optical fibre-based SPR sensors for portable and cost-effective applications.

## Materials and methods

Sensor slides with 50 nm-thick nano-laminated gold film coating were purchased from BioNavis Ltd. The Urea, creatinine, urease and creatininase powder purchased from Sigma Aldrich were used as received. Ultrapure water was used as a buffer from Milipore® system (resistivity of 18.2 MΩ.cm at room temperature of 25°C) and for dilution of urea, creatinine, urease enzyme and creatininase enzyme.

**Fig 1:**
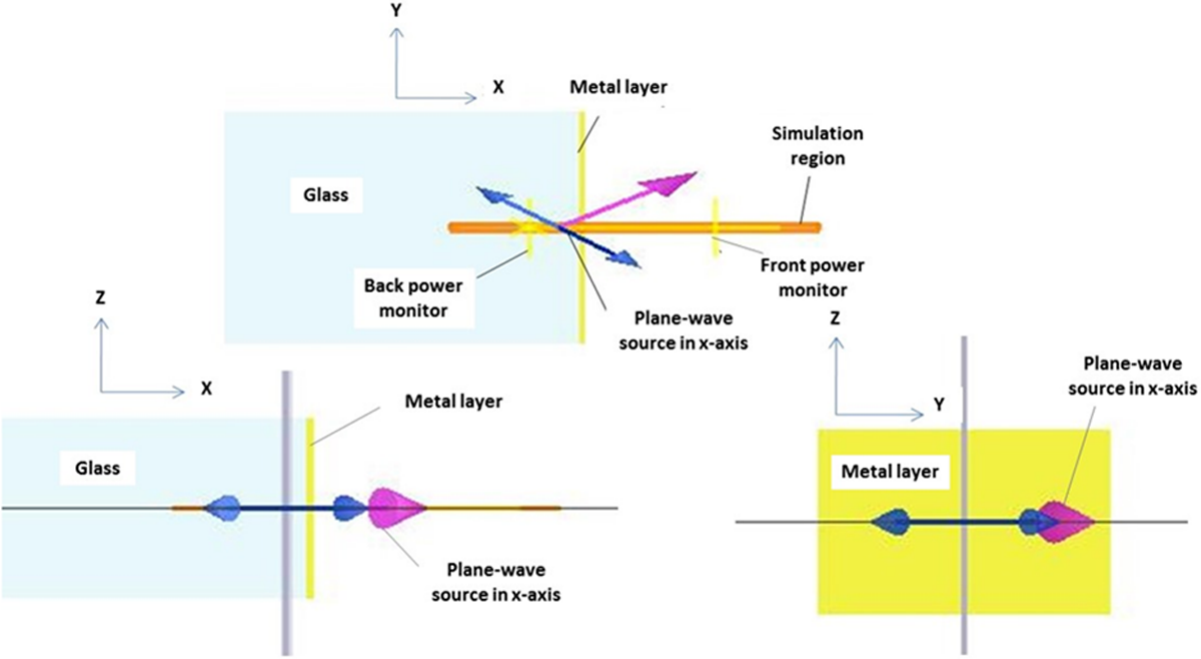
FDTD simulation schematic for the nano-laminated gold thin film on BSG substrate as a SPR-based sensor. The plane-wave source is directed to the metal layer. The back-power monitor placed behind the source measured the reflection from the structure.

Initially, nano-laminated gold film thicknesses suitable for Kretschmann SPR-based sensor were simulated for thicknesses ranging from 20 to 80 nm at optical wavelength of 633 nm using angular interrogation technique. Lumerical’s FDTD software was used for the numerical analysis, where a parameter sweep over the incident angle (36^o^ to 80^o^), was performed in order to obtain the source angle capable to excite the SPR mode. This happens when the *y* component of the source wave vector matches the wave vector of the SPR mode. In other words, the SPR occurs when the wave vector components are parallel to the nano-laminated gold film surface. The perfectly matched layer (PML) profile was set to a "steep angle" to better absorb light propagating at large angles away from the normal incidence. A mesh override region was used to force a finer mesh step size in the *y-*direction which is normal to the metal film. The optical parameter was set as a plane-wave source (Bloch/periodic type) at an optical wavelength of 633 nm. As shown in Fig 1, borosilicate glass (BSG) substrate with refractive index of 1.503 and thickness of 795 nm was used and the simulated dielectric medium was set as air (refractive index, n = 1.00). The Kretschmann configuration in FDTD software using angular interrogation technique shows the XY, XZ and YZ view as in Fig 1 [32]. Further, numerical analysis was conducted with changes of optical parameter for an SPR-based sensor with 50 nm-thick nano-laminated gold film. Angular interrogation technique was applied with variation in the monochromatic wavelengths of 488, 543, 594, 633, 670 and 785 nm.

**Fig 2:**
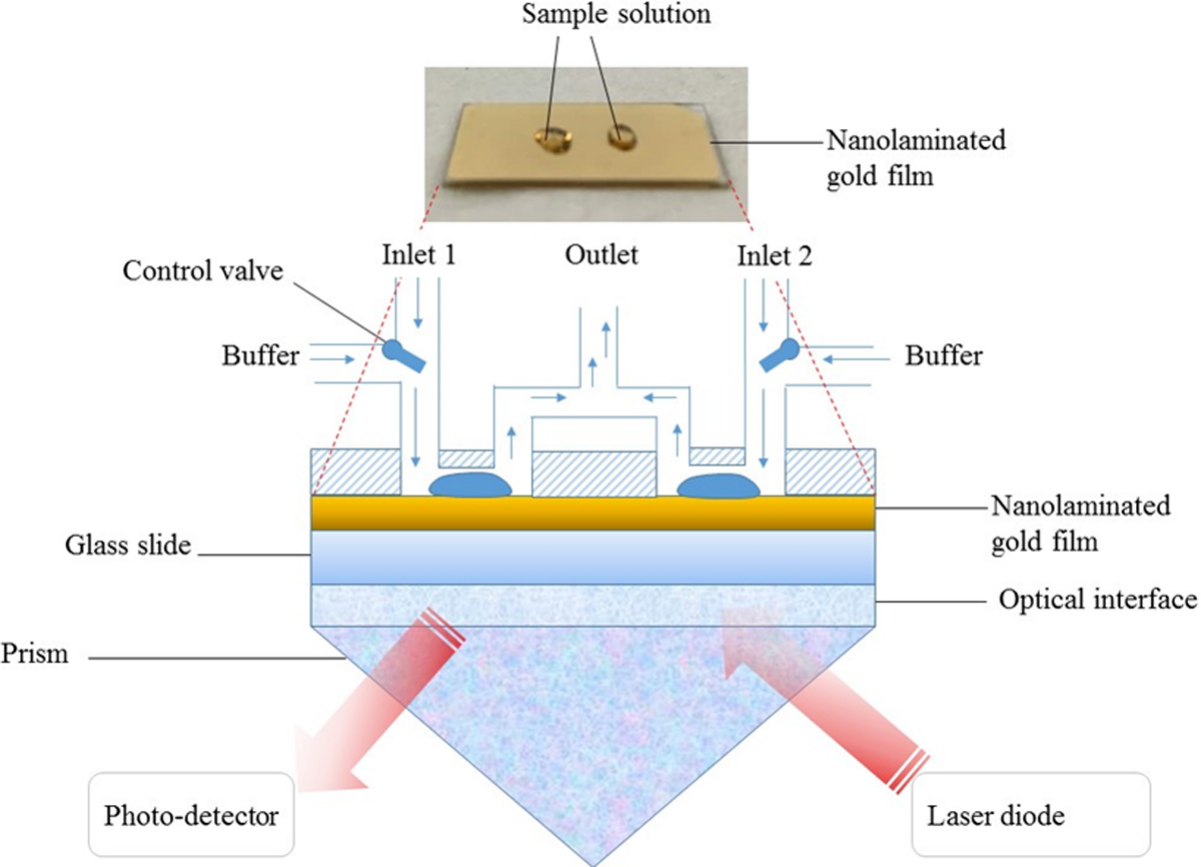
SPR Navi 200 instrument schematic with Kretschmann configuration measurement setup. This instrument’s optics comprises a prism, moving laser diode (wavelength 670 and 785 nm) and a photo-detector, which monitors the intensity of the reflected light as a function of angle. When nanolaminated gold sensor was inserted in the instrument and the flow cell is closed, the glass side of the sensor slide is connected via a refractive index matching elastomer onto the prism, as indicated by optical interface, and the flow cell is connected to the nanolaminate gold film side of the sensor slide.

Next, SPR Navi 200 instrument (BioNavis Ltd.) was used to run the experimental work based on the obtained numerical analysis data. The SPR Navi 200 instrument employed Kretschmann configuration where the incident light is coupled via prism so that the wave number of the incident light is enhanced to match that of the excited surface plasmon. The optical interface between the prism and glass side of the sensor slide contain refractive index matching elastomer to match the refractive index of the prism and the sensor glass slide. Without the use of the refractive index matching elastomer, reflection and refraction might occur at the optical interface and thus the SPR excitation at the gold surface will be affected. As shown in Fig 2, upon exposure to p-polarized light at 670 and 785 nm optical wavelengths, SPR can be excited at the sensor region. As the analyte becomes attached to the surface, the refractive index of the region changes. The sensing region is illustrated by the dielectric layer above the nano-laminated gold film. The changes in refractive index are shown in SPR response curve as shifts in resonance angles. This instrument enables real-time observation of biomolecular interactions, structural changes, and layer properties in both wet and dry state without the labelling process.

Experiments were conducted with various concentrations of urea and creatinine non-enzymatic samples and also enzymatic samples of urea-urease and creatinine-creatininase composition. The non-enzymatic urea samples were prepared by serial dilution with ultrapure water at 100, 200, 400, and 800 mM concentrations, whereas that of creatinine samples were at 10, 20, 50, 100, and 200 mM concentrations. As for the enzymatic samples, urease and creatininase powder were dissolved in ultrapure water based on their molecular weight calculation. The concentration of urease and creatininase enzymes were fixed at 0.682 μmol and 0.05 mmol, respectively. Ideally, the urease enzyme and creatininase enzyme solution were mixed with urea and creatinine solution right before injection into the SPR instrument as the composition denatured easily at room temperature. Accordingly, urease solution (1 ml) was mixed with urea solution (1 ml) from each concentration (100, 200, 400, and 800 mM). Likewise, creatininase solution (1 ml) was mixed with creatinine solution (1 mm) from each concentration (10, 20, 50, 100, and 200 mM).

Prior to the experiment, the peristaltic pump on the SPR instrument was turned on to fill the flow cells with ultrapure water as a buffer at 100 μl/min flow rate. This allows the buffer solution to flow over the nano-laminated gold film. Then, the nano-laminated gold film was exposed to the urea-urease enzymatic sample by injection through inlet 1 while creatinine-creatininase enzymatic sample through inlet 2 using a syringe. The injection valve was kept at ‘inject’ position for two minutes before turning off for another two minutes. The sample solutions flow across the nano-laminated gold film and discharged at outlet. Same procedure was performed on non-enzymatic samples of urea and creatinine solutions at different concentrations.

The reflectivity, sensorgram, and sensitivity analysis of this nano-laminated gold sensor for urea and creatinine detection were measured from the SPR response curves obtained. Sensitivity, *S*_*n*_, is defined as the shift in incident angle per unit change in the concentration of urea and creatinine. It was measured by dividing the shifting angle value (from ultrapure water) per molarity of urea and creatinine solutions and express in (°/M). The SPR response curves were also used to obtain refractive index value of each concentration of urea and creatinine solutions using SPR Navi LayerSolver software. Accordingly, the data from SPR response curves obtained from 670 nm wavelength were used to calculate the thickness (*d*), refractive index (*n*), and dispersion relation (*k*) of the Au thin film under air exposure. Afterward, the refractive index of urea, creatinine, urease, and creatininase solutions at 670 nm were constrained from the linear relationship between λ and *n* (where *n* = *a*+*b*λ).

## Results and discussion

**Fig 3:**
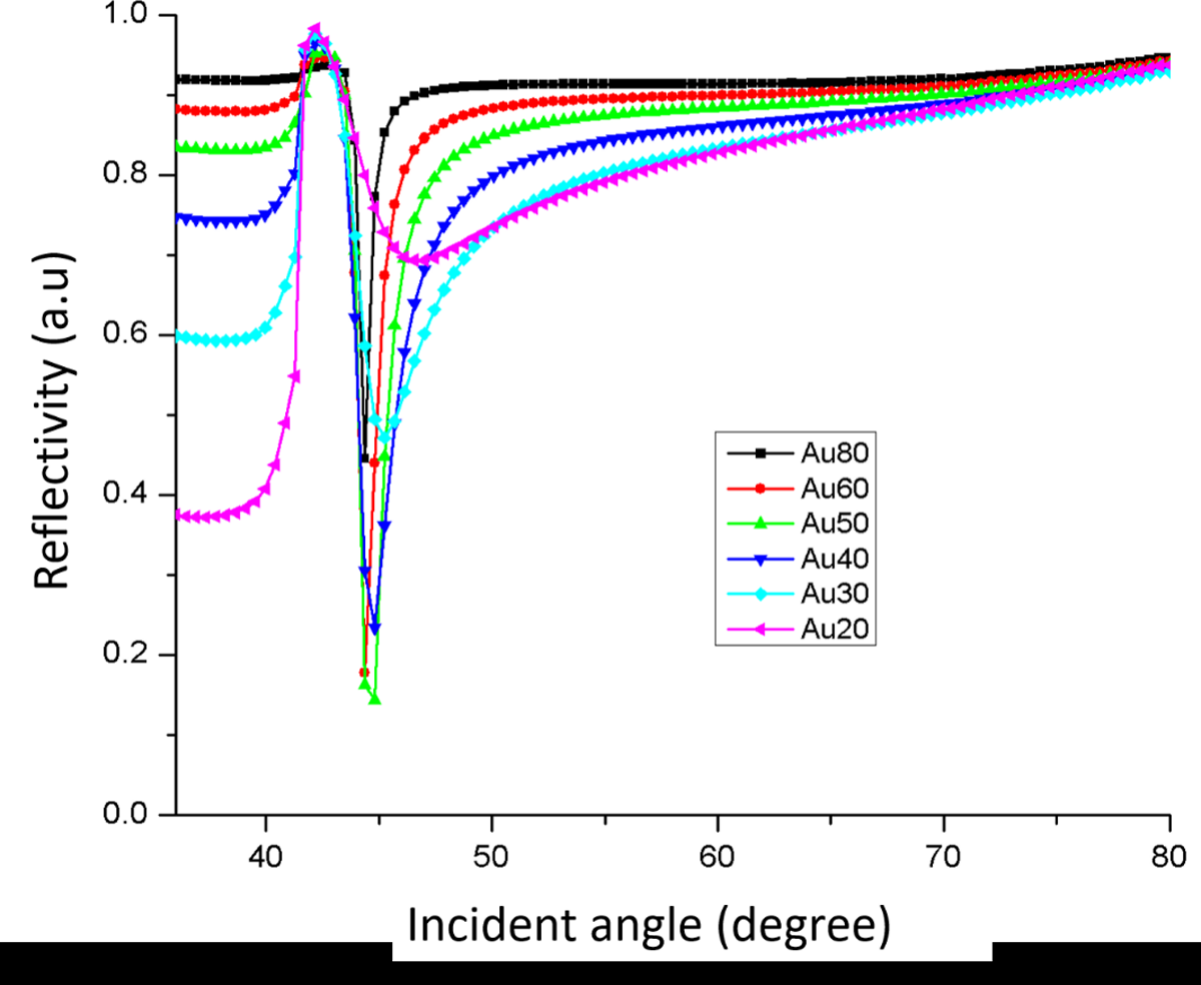
Reflectivity versus incident angle for nano-laminated gold film Kretschmann and SPR-based sensor using FDTD simulation for gold (Au) thin film thickness ranging from 20 to 80 nm.

In order to characterize the behaviour of nano-laminated gold film SPR-based sensor in different thickness, reflectivity was plotted against incident angle in SPR response curves as obtained from the FDTD numerical analysis. Fig 3 shows the reflectivity versus incident angle for nano-laminated gold (Au) thin film thickness ranging from 20 to 80 nm. The dielectric medium was set as air. The results reveal that nano-laminated gold film at 50 nm thickness yield the best reflectivity of 90–92% with a full-width-at-half-maximum (FWHM) of 0.88^o^. When the thickness is below the optimum value, the SPR response curve becomes broad whereas at above the optimum value, it become shallow indicate low reflectivity of the signal.

**Fig 4:**
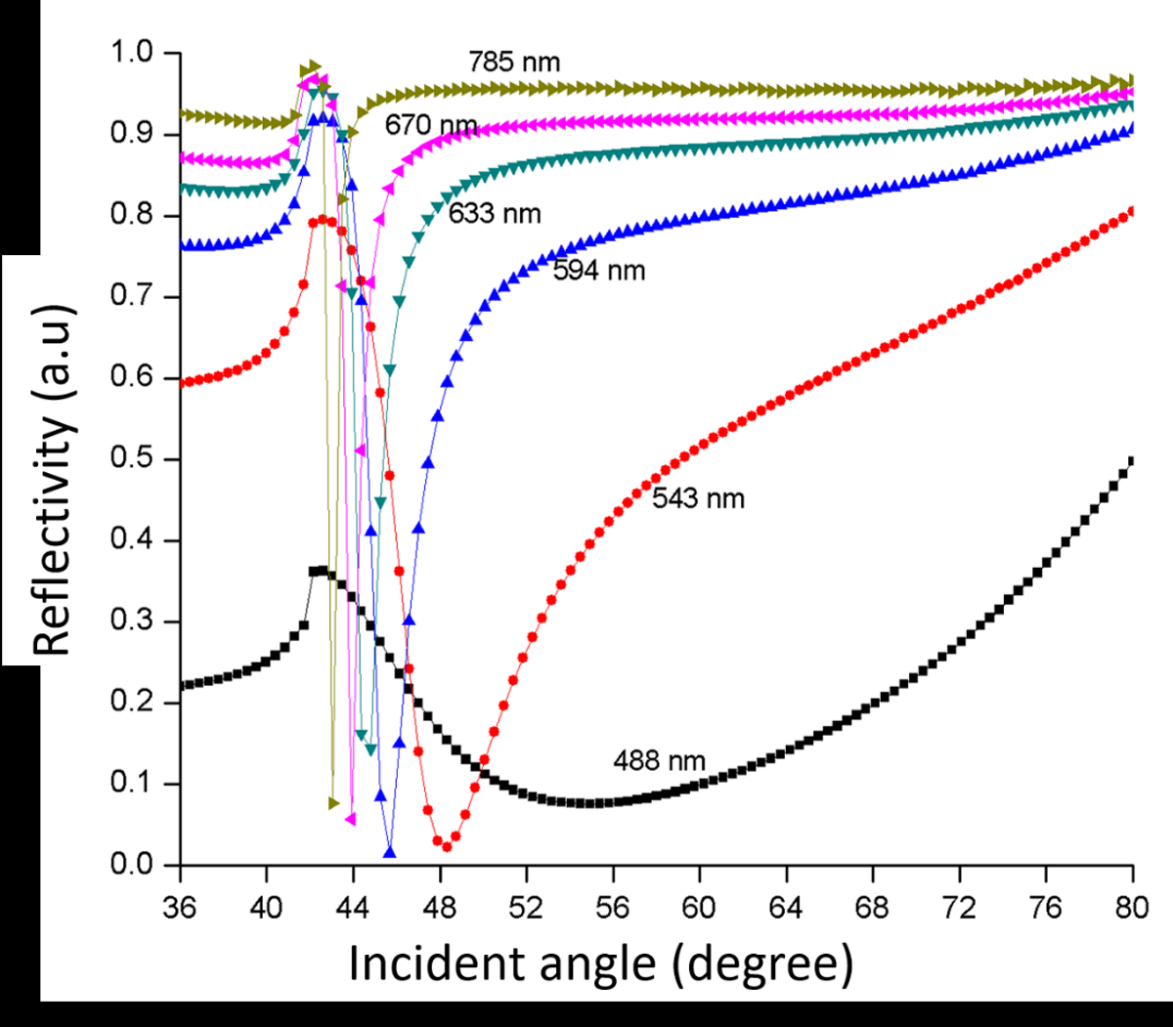
Reflectivity versus incident angle at various wavelengths on SPR sensor slide with nano-laminated gold film.

Further, the numerical analysis conducted in angular interrogation mode with changes of optical parameter for a 50 nm-thick nano-laminated gold film SPR sensor gave plots of reflectivity versus incident angle. Fig 4 shows the SPR response curves of the 50 nm-thick nano-laminated gold film SPR sensor at 488, 543, 594, 633, 670 and 785 nm optical wavelengths. The dielectric medium was set as air. The broad SPR response curve obtained at 488 nm wavelength shows no attenuated total reflection (ATR) occurred. At 543 nm optical wavelength, it gives a resonance angle of 48^o^, which is a higher value compared to 594 nm at 46^o^. Meanwhile, the resonance angle at optical wavelengths of 633, 670 and 785 nm occurred at 45^o^, 44^o^ and 43^o^, respectively. The best SPR response curve occurred at 785 nm wavelength with reflectivity of 90.8% (FWHM = 0.55^o^), followed by 90.2% (FWHM = 0.71^o^) at 670 nm. At the wavelength of 534 nm, reflectivity and FWHM were not measured due to a very shallow and broad curve as a result of poor SPR excitation and it is therefore not suitable for the sensor design.

**Fig 5:**
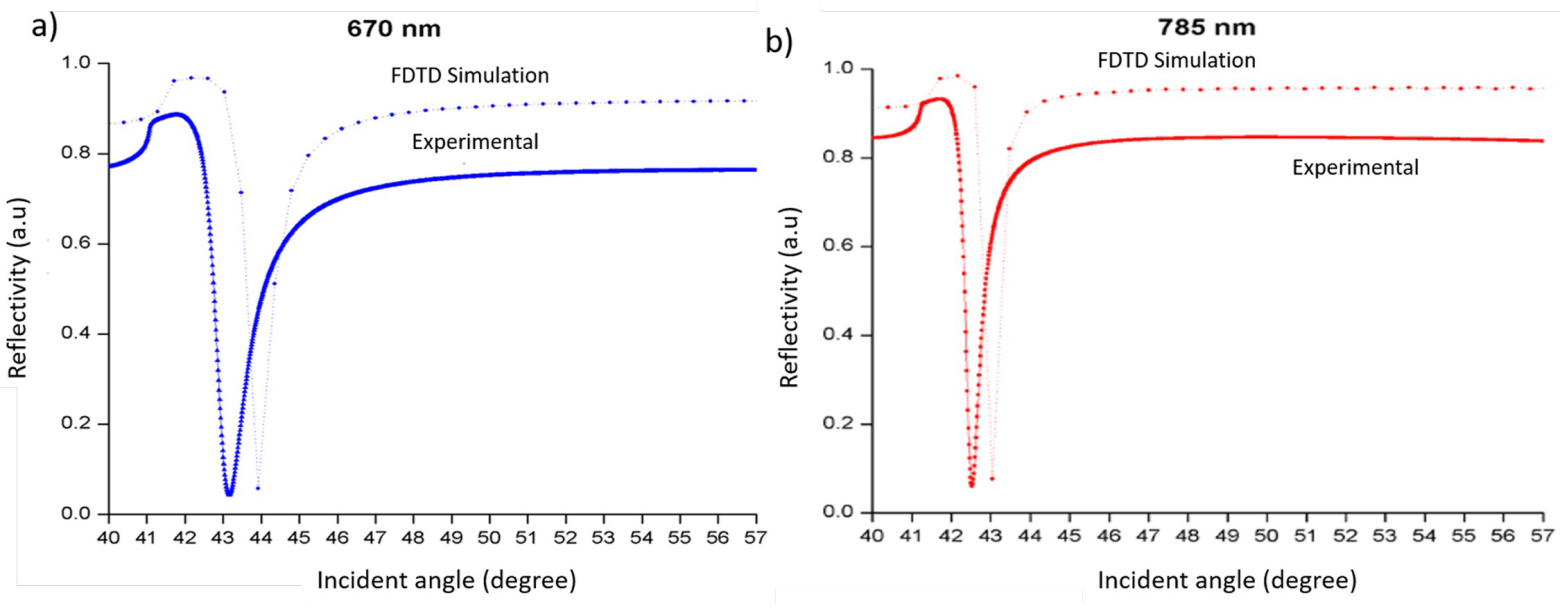
SPR response curve of 50 nm-thick gold nano-laminated film from FDTD simulations and experiments at a) 670 nm and b) 785 nm optical wavelengths.

Fig 5 illustrates the results of experimentally validated numerical analysis on 50 nm-thick nano-laminated gold film as a function of incident angle at optical wavelengths of 670 and 785 nm with air as the dielectric medium. There is a very good agreement between simulation and experimental results with a slight shift to the right of the incident angle (0.5° to 0.8°) and the difference in reflectivity is less than 10%. The small difference might be due to noise factors during experimental measurements such as temperature, pH, humidity, and so forth.

**Fig 6:**
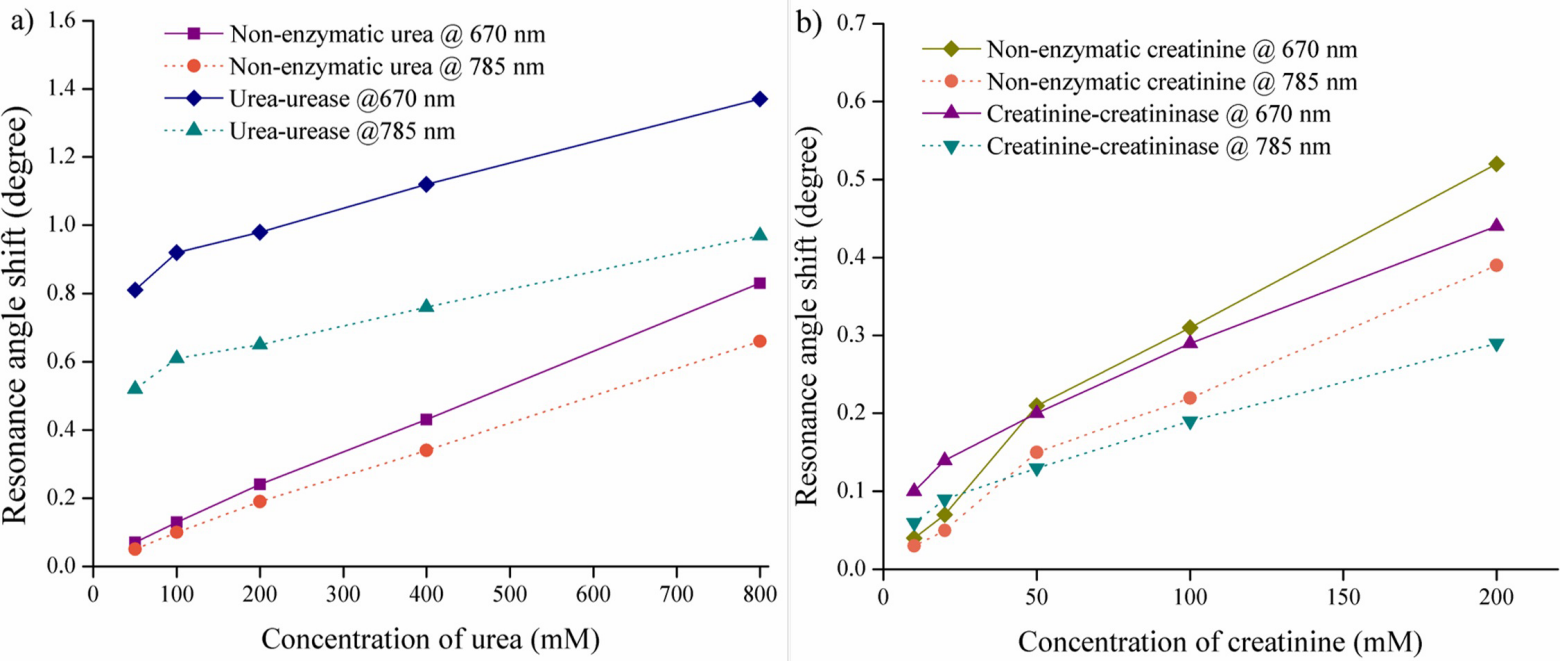
Resonance angle shift of 50 nm-thick nano-laminated gold film sensor towards various concentrations a) urea and b) creatinine at 670 and 785 nm optical wavelengths.

**Fig 7:**
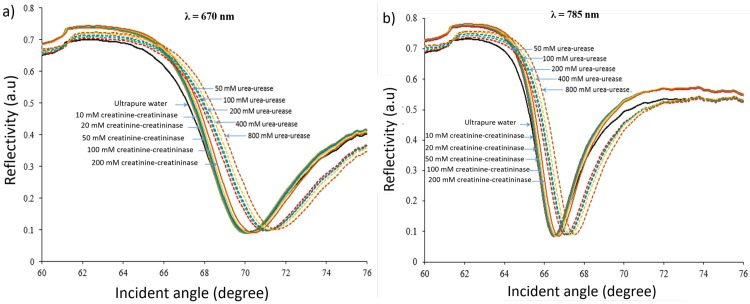
SPR response curves of 50 nm-thick nano-laminated gold film towards various concentrations of urea and creatinine with their enzymes at a) 670 nm and b) 785 nm optical wavelengths.

Next, a double channel SPR Navi 200 instrument in Kretschmann configuration using attenuated total reflection was used for sensing urea and creatinine in experimental studies. A commercially available 50 nm-thick nano-laminated gold SPR sensor was inserted into the sensor holder of the SPR instrument. Flow cell was set close to the triangular prism so that analyte solutions can easily be introduced to flow across the nano-laminated gold film surface. Analysis was undertaken for sensing of urea and creatinine at different concentrations in enzymatic and non-enzymatic samples using 670 nm and 785 nm optical wavelengths. The enzyme urease and creatininase were mix with urea and creatinine solution and were injected into the SPR instrument within one minute in order to prevent the urease and creatininase enzyme degraded. Afterward, the SPR instrument made the measurement and yield a SPR response curve within 30 seconds after the injection. This assure the materials degradation did not occur in this time scale. Fig 6 show the variation of resonance angle shifts with concentration of urea and creatinine in enzymatic and non-enzymatic sample. It can be noted that urea detection in enzymatic sample gave larger resonance angle shift compared to non-enzymatic counterpart at 670 and 785 nm wavelength. Also, there is a linear increase in resonance angle shift as the concentration of urea increased. This is probably due to the coupling activity of urea-urease resulting in increase in refractive index near the sensing surface, and hence the resonance angle shift increased [33]. However, in the creatinine detection at above 40 mM was lower in enzymatic reaction then the non-enzymatic sample. This may be due to limited amount of creatininase to couple with creatinine molecules. As illustrated in Fig 7, a narrower SPR response curve was obtained when sensing urea-urease and creatinine-creatininase enzymatic samples with 785 nm illumination compared to 670 nm. The SPR response curves indicate that this sensor yields better SPR signal at 785 nm optical wavelength, consistent with the results from FDTD simulation. Each of the measurements were repeated three times to check the reproducibility and fluctuation about 4–8% in resonance angle shift was observed. This may be varied due to the environmental factors.

**Fig 8:**
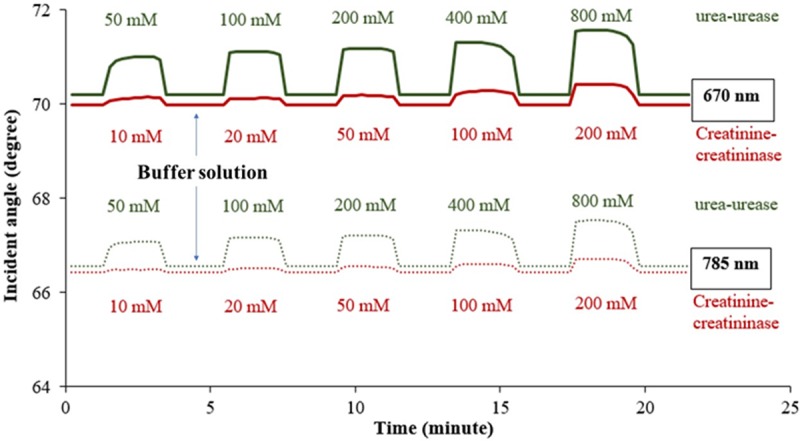
Sensorgram of 50 nm-thick nano-laminated gold with mixed urea-urease and creatinine-creatininase with different concentrations at optical wavelengths of 670 and 785 nm.

In order to observe the SPR response in a clearer manner, a sensorgram of the incident angle shifts versus time for different urea and creatinine concentrations at 670 and 785 nm optical wavelengths were plotted as shown in Fig 8. Ultrapure water was used as buffer solution to obtain the baseline. The curve shows the ‘on-off’ behavior of the SPR biosensor when the nano-laminated gold film was exposed to the buffer solution, urea-urease and creatinine-creatininase enzymatic solution. In the ‘on’ state samples were injected into the SPR instrument through inlet 1 and 2 and flow across the nano-laminated gold film surface, whereas in the ‘off’ state ultrapure water was flow across the sensing surface and the analyte sample was discharged at the outlet. As the concentration of the analytes increased, the resonance angle also increased. The increase in resonance angle was higher in urea-urease samples compared to creatinine-creatininase samples. The sensorgram also shows that the biosensor able to respond promptly (within one minute after sample injection) and continuously, indicating the stability of the equipment and the capability of the nano-laminated gold film to detect different concentrations of urea and creatinine in the presence of the enzymes.

**Fig 9:**
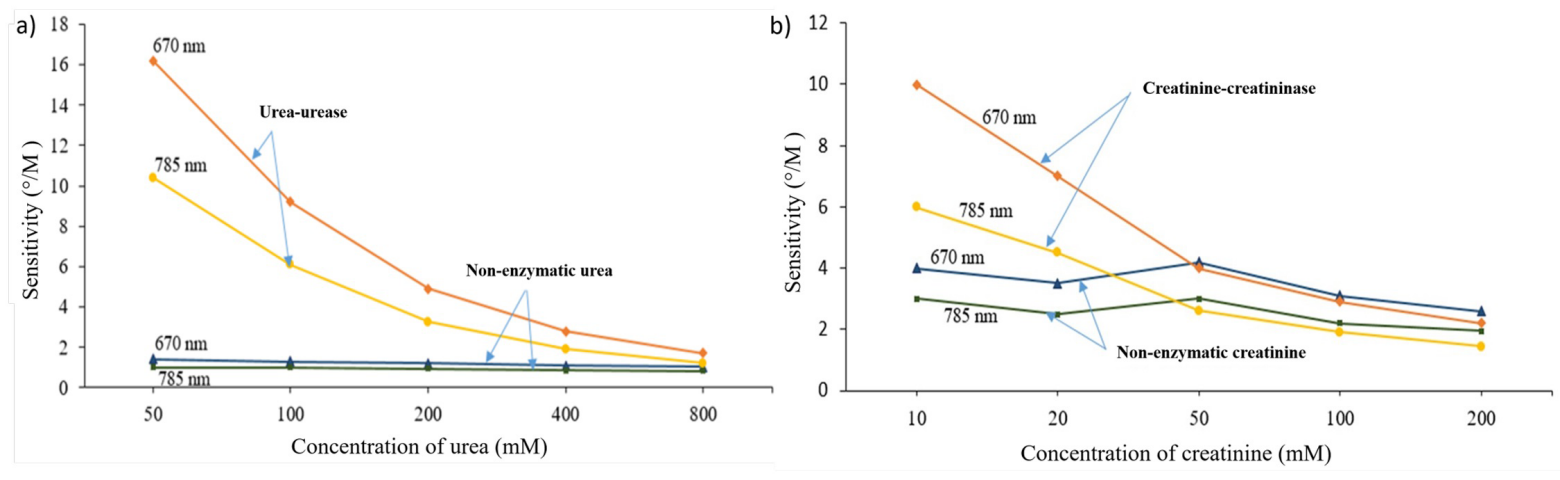
Sensitivity trend of 50 nm-thick nano-laminated gold film with enzymatic and non-enzymatic solutions in various concentrations at optical wavelength of 670 and 785 nm. a) urea-urease and b) creatinine-creatininase.

Results of sensitivity were plotted in sensitivity graph with increment of urea and creatinine concentrations in enzymatic and non-enzymatic samples as shown in Fig 9. For the 50 mM non-enzymatic urea sample, the sensitivity obtained was 1.4°/M at 670 nm and 1°/M at 785 nm. Meanwhile, by comparing the same concentration of non-enzymatic urea sample with the enzymatic urea-urease sample, the latter shows a higher sensitivity of 16.2°/M at 670 nm and 10.4°/M at 785 nm. In the case of creatinine-creatininase sample at 10 mM of creatinine, the sensor exhibits a higher sensitivity of 10°/M at 670 nm compared to 6°/M at 785 nm optical wavelength. However, one can observe that increment in the concentration of urea and creatinine in enzymatic sample resulted in sensitivity reduction. Also, saturation was observed at high concentrations of urea and creatinine with their respective enzymes. This may be due to the limited amount of enzyme molecules at high concentration of urea and creatinine [29]. Thus, the lack of the enzyme molecules at high urea and creatinine concentrations caused reduction in coupling activity and subsequently saturates the biosensor sensitivity. In case of non-enzymatic samples, the sensitivity trend has almost flattened out.

Table 1 shows the list of refractive indices value for non-enzymatic urea and creatinine solutions and enzymatic urea-urease and creatinine-creatininase solutions in different concentration with concentration of urease and creatininase fixed at 0.682 μmol and 0.05 mmol, respectively, on 50 nm-thick nano-laminated gold film sensor at 670 nm optical wavelength. Higher concentrations of urea and creatinine in samples give higher refractive index values of the solution. Based on these data obtained, it can be confirmed that the increase in refractive index resulting in a shift SPR resonance angle.

**Table 1:** Refractive index value of the enzymatic and non-enzymatic solutions in various urea and creatinine concentrations at 670 nm optical wavelength.

**Concentration of urea in non-enzymatic solution (mM)**	**Refractive index, *n***
50	1.33350
100	1.33388
200	1.33397
400	1.33542
800	1.33739
**Concentration of urea in enzymatic urea-urease solution (mM)**	**Refractive index, *n***
50	1.33433
100	1.33525
200	1.33561
400	1.33654
800	1.33790
**Concentration of creatinine in non-enzymatic solution (mM)**	**Refractive index, *n***
10	1.33295
20	1.33297
50	1.33305
100	1.33311
200	1.33320
**Concentration of creatinine in enzymatic creatinine-creatininase solution (mM)**	**Refractive index, *n***
10	1.33300
20	1.33303
50	1.33307
100	1.33314
200	1.33328

It is demonstrated that the enzymatic samples on nano-laminated gold film SPR-based sensor had increased in sensitivity compared to the non-enzymatic samples. The improved sensitivity of the sensor was due to the increased in refractive index of analyte sensing layer as a result of urea-urease and creatinine-creatininase coupling activity. The presence of urease and creatininase enzymes in samples served as biomolecules recognition molecule in the sensor system. This proved that the Kretschmann-based SPR sensor with nano-laminated gold film for urea and creatinine detection using enzymatic solution is indeed a suitable method, especially for artificial kidney applications. It is an economical and label-free detection method with ease of operation, excellent sensitivity, and rapid detection in designing urea and creatinine sensor which will subsequently lead to a portable optical fibre-based SPR sensor applications. This work can be a proof-of-concept experiment and it can be applied to other analyte such as glucose and uric acid. The selectivity study using nano-laminated gold film sensor with enzyme immobilized on the sensing layer will be conducted in our future study with real sample from haemodialysis effluent. Immobilizing the enzyme on the sensor surface allows reusable usage of enzyme to for analyte detection. In this way the cost for operating the Kretschmann based SPR biosensor can be further reduced.

## Conclusion

We have successfully investigated a nano-laminated gold film SPR-based sensor using Kretschmann configuration for urea and creatinine detection. To the best of our knowledge, this is a novel investigation on the SPR sensitivity of 50 nm-thick nano-laminated gold film towards the different concentration of urea and creatinine in the presence of their respective enzymes using Kretschmann configuration. The sensorgram and sensitivity of the nano-laminated gold film towards the enzymatic and non-enzymatic sample of various concentrations have been successfully investigated and analysed. The effects of couple activity of analyte-enzyme in the enzymatic samples were believed to enhance the sensitivity of the sensor by ~150 to 1057% compared to a non-enzymatic solution (at 670 nm wavelength). Additional advantages of this sensor include low cost, commercial availability of nano-laminated gold film and capability of detecting even a small increase in urea and creatinine concentrations.
